# The Long-Term Outcomes of Corticosteroid Use in COVID-19 Patients with Cardiovascular Disease: A Propensity-Matched Analysis from the Multi-Center International Prospective Registry (HOPE-2)

**DOI:** 10.3390/biomedicines13112665

**Published:** 2025-10-30

**Authors:** Jorge García-Onrubia, Ravi Vazirani, Gisela Feltes, Rafael Sánchez-Del Hoyo, María C. Viana-Llamas, Sergio Raposeiras-Roubín, Rodolfo Romero, Emilio Alfonso-Rodríguez, Aitor Uribarri, Francesco Santoro, Víctor Becerra-Muñoz, Martino Pepe, Alex F. Castro-Mejía, Jaime Signes-Costa, Adelina Gonzalez, Francisco Marín, Javier Lopez-País, Enrico Cerrato, Olalla Vázquez-Cancela, Carolina Espejo-Paeres, Álvaro López Masjuan, Lazar Velicki, Ibrahim El-Battrawy, Harish Ramakrishna, Antonio Fernandez-Ortiz, Ivan J. Nuñez-Gil

**Affiliations:** 1 Department of Cardiology, Cardiovascular Institute, Hospital Clínico San Carlos, Instituto de Investigación Sanitaria del Hospital Clínico San Carlos (IdISSC), 28040 Madrid, Spain; jorgegonrubia@gmail.com (J.G.-O.); ravi.vazirani@salud.madrid.org (R.V.);; 2 Cardiology Department, Hospital Vithas Arturo Soria, 28043 Madrid, Spain; 3 Department of Cardiology, Hospital Universitario de Torrejón, 28850 Torrejón, Spain; 4 Faculty of Biomedical and Health Sciences, Universidad Europea de Madrid, 28670 Madrid, Spain; 5 Research Methodological Support Unit and Preventive Department, Hospital Clínico San Carlos, Instituto de Investigación Sanitaria del Hospital Clínico San Carlos (IdISSC), 28040 Madrid, Spain; 6 Cardiology Department, Hospital Universitario de Guadalajara, 19002 Guadalajara, Spain; 7 Cardiology Department, Hospital Universitario Álvaro Cunqueiro, 36312 Vigo, Spain; 8 Emergency Department, Hospital Isabel Zendal, Hospital Universitario de Getafe, 28905 Madrid, Spain; 9 Cardiology Department, Bellvitge Biomedical Research Institute (IDIBELL), Hospital Universitario deBellvitge, 08908 Barcelona, Spain; 10 Cardiology Department, Hospital Univesitari General de Catalunya, 08195 Barcelona, Spain; 11 Cardiology Department, Hospital Universitari Vall d’Hebron, Centro de Investigación Biomédica en Red de Enfermedades Cardiovasculares (CIBERCV), 08035 Barcelona, Spain; 12 Cardiology Department, Ospedali Riuniti, 71122 Foggia, Italy; 13 Cardiology Department, Hospital Clínico Universitario Virgen de la Victoria, Centro de Investigación Biomédica en Red de Enfermedades Cardiovasculares (CIBERCV), 29010 Malaga, Spain; 14 Division of Cardiology, Department of Interdisciplinary Medicine (D.I.M.), University of Bari Aldo Moro, 70121 Bari, Italy; 15 Hospital General del Norte de Guayaquil IESS “Los Ceibos”, Universidad Espíritu Santo, Guayaquil 090615, Ecuador; 16 Pneumology Department, Hospital Clínico de Valencia, Instituto de Investigación Sanitaria (INCLIVA), University of Valencia, 46010 Valencia, Spain; 17 Anesthesiology Department, Hospital Universitario Infanta Sofia, 28702 Madrid, Spain; 18 Cardiology Department, Hospital Clínico Universitario Virgen de la Arrixaca, IMIB-Arrixaca, Centro de Investigación Biomédica en Red de Enfermedades Cardiovasculares (CIBERCV), 30120 Murcia, Spain; 19 Cardiology Department, Hospital Clínico Universitario de Santiago de Compostela, 15706 Santiago de Compostela, Spain; 20 Cardiology Department, San Luigi Gonzaga University Hospital, Orbassano and Rivoli Infermi Hospital, 10098 Rivoli, Italy; 21 Preventive Department, Hospital Universitario de Santiago de Compostela, 15706 Santiago de Compostela, Spain; 22 Cardiology and Emergency Department, Hospital Universitario Príncipe de Asturias, 28805 Madrid, Spain; 23 Cardiology Department, Hospital Universitario Juan Ramón Jimenez, 21005 Huelva, Spain; 24 Faculty of Medicine, University of Novi Sad, 21000 Novi Sad, Serbia; 25 Institute of Cardiovascular Diseases Vojvodina, 21204 Sremska Kamenica, Serbia; 26 Institute of Physiology, Department of Cellular and Translational Physiology, Medical Faculty, Ruhr University of Bochum, 44801 Bochum, Germany; 27 Institut für Forschung und Lehre (IFL), Molecular and Experimental Cardiology, Ruhr University of Bochum, 44801 Bochum, Germany; 28 Department of Cardiology, St. Josef-Hospital of the Ruhr University Bochum, 44801 Bochum, Germany; 29 Anesthesiology Department, Mayo Clinic, Rochester, MN 55905, USA

**Keywords:** SARS-CoV-2 infection, corticosteroid therapy, pre-existing heart disease, prognosis

## Abstract

**Introduction**: Corticosteroid therapy has been demonstrated to improve prognosis and reduce mortality in patients with severe Coronavirus Disease 2019 (COVID-19) infection by attenuating the exaggerated inflammatory response that emerges in the late phase of infection. However, its impact on patients with pre-existing cardiovascular disease, who are at higher risk of complications, has not been specifically studied. The aim of this study is to evaluate the effect of corticosteroid therapy on mortality and long-term COVID-19 symptoms in this high-risk population. **Methods**: We analyzed the prospective registry HOPE-2. Patients with previous cardiovascular disease were selected, and 18-month all-cause death was defined as the primary endpoint. Long-term COVID-19 symptoms were considered as secondary endpoints. A total of 1188 patients with previous heart disease were included, of which 453 received corticosteroid treatment. Propensity score matching analysis in a 1:1 fashion was performed based on baseline variables that exhibited a *p*-value < 0.05 in the univariant analysis and outcome variables that defined corticosteroid use, with a final matched population of 796 patients. **Results**: In patients with pre-existing heart disease, corticosteroid treatment was not associated with differences in 18-month all-cause mortality (*p* = 0.52). However, a shorter duration of hospitalization (median: 8 days [IQR: 4–14] and 11 days [IQR: 7–18]; *p* < 0.001) was observed in patients who received corticosteroids. No significant differences in long-term COVID-19 symptoms were observed between the two groups. **Conclusions**: In patients with pre-existing heart disease, the absence of a clear harmful effect suggests that the positive effects of corticosteroids may be offset by their potential adverse effects which could contribute to the persistence of long COVID symptoms. This finding may reflect a differential response to corticosteroids in this high-risk subgroup, highlighting the need for further studies to clarify the role of this therapy in such patients.

## 1. Introduction

The novel coronavirus Severe Acute Respiratory Syndrome Coronavirus 2 (SARS-CoV-2), which emerged from a zoonotic source, was initially identified in December 2019, leading to the declaration of the Coronavirus Disease 2019 (COVID-19) pandemic by the World Health Organization on 11 March 2020 [1].

While many infected individuals experience mild or no symptoms, a substantial proportion of patients develop severe disease requiring hospitalization, particularly those with underlying cardiovascular conditions [2].

In the RECOVERY study, patients hospitalized due to COVID-19 were allocated to receive either dexamethasone or standard care, with 28-day mortality as the main endpoint. Treatment with dexamethasone significantly reduced death among individuals who required invasive ventilation or supplemental oxygen, whereas no survival benefit was seen in those who did not need respiratory support or who were given higher doses [1]. Matthay et al. suggested that corticosteroid use at early disease stages or at high cumulative doses could be linked to poorer outcomes [3], a concept later examined in several observational cohorts [4,5].

Evidence of cardiac injury, reflected by increased circulating troponin concentration during COVID-19 infection, has been associated with unfavorable prognosis, particularly among individuals with prior cardiovascular comorbidities [6].

In addition, a retrospective analysis of individuals admitted for pneumonia reported that exposure to systemic corticosteroids correlated with a higher incidence of significant cardiovascular complications, especially in patients with cardiovascular risk factors or in those receiving higher doses or prolonged treatments [7].

**Figure 1:**
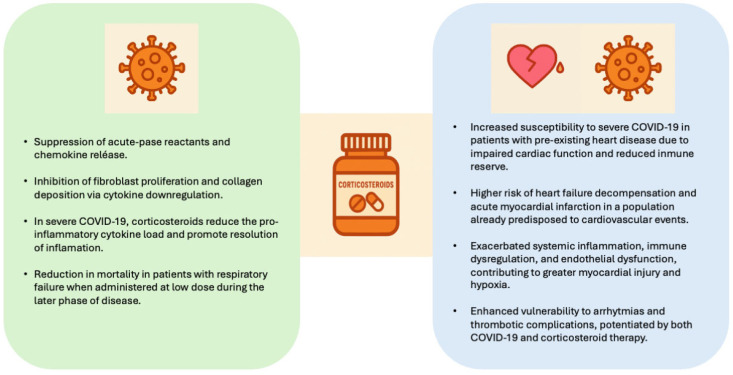
Effects of corticosteroids in COVID-19 patients with and without pre-existing heart disease.

Prior studies have shown that individuals with pre-existing cardiovascular disorders experience worse clinical outcomes and are more likely to develop persistent sequelae following SARS-CoV-2 infection. These patients exhibit both higher in-hospital and post-discharge mortality rates and report more frequent long-term symptoms after recovery. The adverse prognosis seems to stem from an exaggerated inflammatory response, dysregulated immune signaling, and endothelial impairment, which may intensify myocardial damage and tissue hypoxia [8,9]. Despite these associations, the effect of corticosteroid therapy in this population has not been clearly determined, and its clinical value continues to be debated. Figure 1 illustrates the effects of corticosteroid therapy in COVID-19 patients according to the presence or absence of pre-existing heart disease.

This study sought to analyze how corticosteroid treatment for COVID-19 influenced clinical outcomes in patients with pre-existing cardiovascular disease and to explore its potential association with persistent post-COVID-19 symptoms in a prospective multi-center registry.

## 2. Materials and Methods

### 2.1. Study Design and Participation Criteria

The HOPE-2 registry (Health Outcome Predictive Evaluation for COVID-19—2) [NCT04778020] is a prospective, investigator-initiated, multi-center study conceived under an “all-comers” approach and carried out without financial remuneration for the participating researchers. The dataset analyzed in this work can be obtained from the corresponding author upon reasonable request. Inclusion criteria encompassed all patients admitted to any participating hospital with either confirmed COVID-19 or a high clinical suspicion of infection, irrespective of survival status. COVID-19 was confirmed through nasopharyngeal or oropharyngeal swab samples processed by a real-time reverse transcriptase polymerase chain reaction (RT-PCR) according to World Health Organization recommendations. For patients who died before testing, infection was considered confirmed when there was a high clinical suspicion of infection. No specific exclusion criteria were applied except for explicit refusal to participate. Clinical management and decision-making were performed by attending physicians in accordance with each institution’s local protocols and international organizations recommendations that were in effect during the study period [8,10].

The study protocol received ethical approval from the Ethics Committee of Hospital Clínico San Carlos (reference 21/128-E) and was subsequently endorsed by the ethics committees or institutional review boards of all participating sites. Given its anonymized and observational design, the coordinating ethics committee granted a waiver of written informed consent, in line with Spanish law and international ethical principles for health-related research. Data were collected electronically through a secure online platform, starting on 15 March 2021, and the present manuscript includes analyses performed up to 31 December 2021. Data integrity and manuscript validation were overseen by the principal investigators at each center. Detailed study definitions and the full list of collaborators have been reported previously [8].

### 2.2. Data Acquisition and Study Definitions

Eligible participants were those with a documented history of cardiovascular disease who experienced either confirmed or clinically suspected COVID-19 infection during any hospital admission. The presence and type of cardiac disease were identified from medical records or ongoing treatments and were pragmatically classified by site investigators as arrhythmic, ischemic, heart failure or cardiomyopathy, valvular, combined or unspecified, and other forms such as congenital heart disease [8].

**Figure 2:**
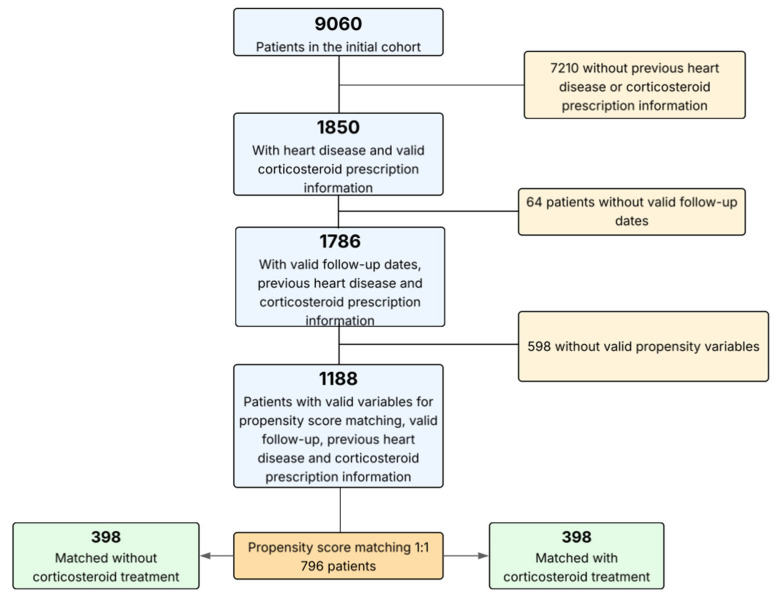
A flowchart with a stepwise description of the selection process.

The administration of corticosteroids was conducted at the discretion of the attending clinicians, guided by the patient’s condition and indications such as pneumonia, sepsis, systemic inflammatory response syndrome, requirement for invasive or non-invasive ventilation, circulatory or extracorporeal membrane oxygenation (ECMO) support, or high-flow oxygen therapy. Outcomes during follow-up were documented locally according to predefined registry criteria. The overall patient selection process is illustrated in Figure 2.

### 2.3. Study Follow-Up and Outcomes

The present analysis included hospitalized individuals with confirmed COVID-19 infection and a documented history of cardiovascular disease. The primary outcome was all-cause mortality at 18 months, whereas secondary endpoints addressed the occurrence of long-term post-COVID-19 symptoms. Follow-up data were gathered through standardized telephone assessments conducted with patients or their relatives and were complemented by information provided by attending clinicians and reviews of medical records.

### 2.4. Statistical Analysis

Continuous variables were summarized as the mean ± standard deviation when normally distributed or as the median with the interquartile range otherwise. Categorical variables were expressed as counts and percentages. Comparisons between groups were performed with Student’s *t*-test or the Mann–Whitney U test for continuous variables and with Pearson’s chi-square or Fisher’s exact test for categorical variables.

Within the subgroup of patients presenting pre-existing cardiac disease, a propensity score for corticosteroid treatment was constructed based on baseline variables showing a *p*-value < 0.05 in the univariable analysis (including renal impairment, chronic pulmonary disease, and immunosuppression). The model was additionally adjusted for clinical factors defining severe SARS-CoV-2 and potential indication for corticosteroid therapy (pneumonia, sepsis, respiratory failure, and systemic inflammatory response syndrome [SIRS]). Subsequently, a 1:1 matched sample was generated by pairing treated and untreated individuals using nearest-neighbor matching with a caliper width of 0.5.

Survival was assessed through Kaplan–Meier curves with log-rank testing. The survival period was calculated from the date of hospital admission to the 18-month follow-up. Mortality comparisons were carried out with chi-square testing. A post hoc sensitivity analysis using the log-rank test was performed as well to explore a possible association between all-cause death and invasive mechanical ventilation as well as statin therapy in the post-PSM population. The length of hospital stay was analyzed with the Mann–Whitney U test.

All statistical analyses were conducted with IBM SPSS Statistics v26.0 (SPSS Inc., Chicago, IL, USA) and R v4.4.1. Two-tailed tests were applied, and a *p*-value < 0.05 was considered statistically significant. A pre-specified sensitivity analysis was also performed to assess whether the timing of corticosteroid initiation (within ±7 days of symptom onset) influenced overall mortality.

## 3. Results

The baseline clinical profile of patients with pre-existing heart disease who were hospitalized with or without corticosteroid therapy is summarized in Supplementary Table S1. Individuals receiving corticosteroids more frequently presented a history of chronic lung disease, chronic kidney disease, or immunosuppression and were less often treated with statins. During hospitalization, these patients exhibited a higher incidence of pneumonia, respiratory failure, and systemic complications such as sepsis, acute kidney injury, and systemic inflammatory response syndrome (Table S1).

The clinical characteristics of both groups after propensity score matching on baseline and outcome variables are detailed in Table 1.

In the matched cohort, patients with corticosteroid treatment experienced a higher frequency of in-hospital bleeding events and the need for invasive mechanical ventilation. Additionally, the proportion of individuals on statin therapy remained higher among those treated with corticosteroids.

Table 2 shows the distribution of the different types of heart disease in the matched population. No significant differences were found in the distribution of heart disease types between the two groups (*p* = 0.83).

**Figure 3:**
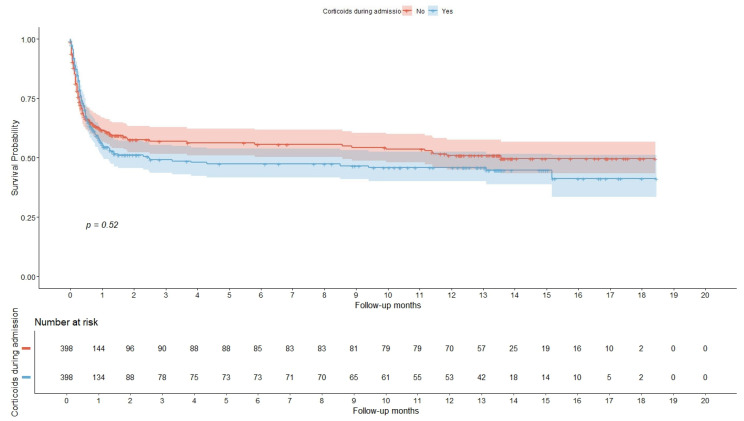
Survival analysis represented by Kaplan–Meier curves (**upper part**) and number of patients at risk after end of each time (**lower part**). Shaded area surrounding each curve represents 95% confidence interval.

Survival analysis is presented by Kaplan–Meier curves in Figure 3, demonstrating no differences in all-cause mortality at 18 months in the log-rank test (*p* = 0.52) in patients with previous heart disease treated with corticosteroids compared to those without corticosteroid treatment. The comparison of hospital stay and categorical mortality analysis (Table 1) indicated a shorter length of hospitalization in the corticosteroid group, while overall mortality remained similar. In-hospital mortality, assessed through a chi-squared test, also revealed no statistically significant differences between the two treatment groups.

We conducted a pre-specified sensitivity analysis to assess whether the timing of corticosteroid initiation had a significant influence on all-cause mortality, and no significant differences were found. Additionally, we performed a post hoc sensitivity analysis to assess the association between all-cause death and invasive mechanical ventilation in the matched population, as well as statins. We generated Kaplan–Meier curves for patients with and without each of the aforementioned therapies, comparing survival according to corticosteroid use in both subgroups, with no significant differences observed.

A comparison of long-term post-COVID-19 symptoms across groups is summarized in Table 3. No significant relationship was observed between corticosteroid therapy and the occurrence of persistent symptoms in patients with pre-existing cardiac disease, except for major bleeding events, which were more frequent among corticosteroid users. The number of reports of dizziness tended to be higher in the steroid-treated group, whereas myalgia was more common among those who did not receive corticosteroids, although these differences did not reach statistical significance.

## 4. Discussion

In this study, we evaluate the effect of corticosteroid treatment in patients with previous heart disease who were hospitalized with a COVID-19 infection on mortality and long-term COVID-19 symptoms.

The main findings in terms of patients with previous heart disease are as follows: (1) Systemic corticosteroid use was not associated with a higher all-cause death rate at 18 months. (2) Patients who underwent corticosteroid treatment had a shorter in-hospital stay. (3) No association was found between corticosteroid treatment and long-term COVID-19 symptoms.

Several prior investigations have characterized the distinctive clinical features of COVID-19 infection among individuals with pre-existing cardiac disease [1], and others have suggested that corticosteroid therapy may exert harmful cardiovascular effects [10,11]. However, no previous study has specifically examined the impact of corticosteroid use in patients with underlying heart disease and COVID-19 infection. The present work aimed to determine whether the response to corticosteroid treatment differs in this subgroup of patients with underlying heart disease.

The mechanisms underlying myocardial involvement likely include the direct viral invasion of cardiomyocytes together with indirect contributors such as hypoxia-related damage, local inflammatory activity, and increased prothrombotic signaling [1]. Angiotensin-converting enzyme 2 (ACE2), which serves as the binding receptor for the viral spike protein, has been shown to be upregulated in cardiac tissue from patients with heart failure, prior myocardial infarction, and diabetes [12,13,14].

Although a mortality benefit of corticosteroid treatment has been observed in the general population, this study did not find a significant association between corticosteroid treatment and mortality in our cohort of patients with heart disease.

Statin therapy has previously been associated with a favorable prognosis in the context of COVID-19 infection [15]. In our cohort, the use of statins was more frequent among patients who did not receive corticosteroid therapy. This finding could be related to a higher prevalence of comorbidities in this subgroup, having led clinicians to be more cautious or reluctant to prescribe corticosteroids due to concerns about potential adverse effects in fragile patients. However, after further analysis, this imbalance did not influence the association between steroid treatment and mortality.

Corticosteroid use has been related to several unfavorable cardiovascular outcomes. Observational data indicate that oral corticosteroid therapy may elevate the risk of acute myocardial infarction, particularly within the first month of exposure, and has also been associated with the onset of atrial fibrillation [16,17]. Among patients hospitalized for pneumonia, systemic corticosteroid administration has been correlated with a higher rate of cardiovascular complications, with the likelihood increasing proportionally to both treatment duration and cumulative dose [7].

Glucocorticoid therapy is recognized as elevating blood pressure, an effect that frequently manifests soon after treatment initiation [18]. Although the precise mechanisms remain incompletely clarified, available data point toward an imbalance between vasoconstrictive and vasodilatory responses, mediated by alterations in vasoactive mediators, oxidative stress, and the stimulation of the renin–angiotensin system [17]. Moreover, corticosteroids promote renal sodium and water retention, potentially worsening fluid overload and contributing to adverse outcomes in patients with heart failure [19]. Consistent with volume overload, several studies examining the link between glucocorticoid exposure and cardiovascular risk have described stronger associations with heart failure than with other cardiac complications [20,21,22].

In our matched analysis, corticosteroid administration was significantly linked to a higher incidence of in-hospital bleeding events. This observation may reflect the well-documented association between corticosteroid exposure and an elevated risk of gastrointestinal hemorrhage [23]. Although the underlying mechanism has not been fully elucidated, corticosteroids are thought to interfere with tissue regeneration, potentially delaying mucosal healing [23]. Prophylactic anticoagulation during the acute phase of COVID-19 has been reported to reduce 30-day mortality in observational cohorts [24]. Additionally, Santoro et al. demonstrated that the combination of aspirin and prophylactic anticoagulation conferred a lower mortality risk compared with anticoagulation alone in hospitalized COVID-19 patients, suggesting a synergistic antithrombotic effect [25]. While these interventions appear to improve short-term prognosis, they simultaneously increase bleeding susceptibility. In our study, the distribution of these therapies was comparable between groups, supporting a potential contribution of corticosteroid therapy itself to the observed excess of bleeding events.

Interestingly, we observed an increased risk of clinically relevant bleeding during follow-up among individuals who received corticosteroids in the acute phase of infection. Yao et al. reported that hemorrhagic complications related to short corticosteroid “bursts” peak during the first month after exposure and, although they decline over time, remain slightly elevated for up to 90 days after therapy initiation [26]. This temporal pattern may account for the greater number of bleeding episodes identified in the corticosteroid-treated subgroup during follow-up.

These pathophysiological mechanisms, when acting in a population with pre-existing heart disease, where the baseline risk of such complications is already elevated, may partly explain why corticosteroid treatment did not confer a survival benefit in our cohort of patients with underlying cardiovascular disease. The lack of a statistically significant relationship between steroid use and mortality emphasizes the need for additional research focused on this specific population.

Nevertheless, we did not observe detrimental cardiovascular consequences among corticosteroid-treated patients, as long-term mortality was comparable between groups. Interestingly, corticosteroid treatment was associated with a shorter duration of hospitalization, suggesting a potential advantage in recovery time and clinical stabilization, as noted in previous reports [2,27,28].

Long-term COVID-19 symptoms are common, particularly in patients who were hospitalized or have a history of heart disease [29]. Such manifestations tend to occur more frequently and resolve more slowly in individuals with cardiovascular comorbidities [30]. Freund et al. linked the severity of acute infection with greater respiratory symptom burden and reduced diffusing capacity (DLCO) at three-month follow-up, reinforcing the role of acute disease severity in the development of long-term sequelae [31].

Although some studies have suggested that corticosteroid therapy during the acute phase of COVID-19 may lower the risk of post-COVID-19 syndrome, our results did not confirm this benefit in patients with underlying heart disease [32].

Corticosteroid therapy has been linked to a higher likelihood of developing long-term cardiovascular complications related to COVID-19, including hypertension, acute coronary syndromes, and atrial fibrillation [17]. In our cohort, however, the frequency of these events during follow-up was comparable between steroid-treated patients and those who did not receive such therapy. This finding may be explained by the overall low incidence of cardiovascular events, which could have reduced the statistical power to identify differences, and by the short-lived nature of corticosteroid-induced cardiovascular effects. Furthermore, the slower recovery trajectory typical of cardiovascular patients may obscure potential differences between treatment groups.

### Clinical Implications

Our study tried to analyze the clinical response to corticosteroid therapy in COVID-19 patients with previous heart disease. We found an association between corticosteroid use and shorter hospital stay, likely related to the impact of these drugs on symptom duration. However, no clear association was demonstrated with long-term symptoms or reduced in-hospital or 18-months mortality.

Although corticosteroid therapy is generally advised for patients with COVID-19-related respiratory failure who lack prior cardiac disease, its administration in individuals with established heart conditions remains complex due to the potential adverse cardiovascular effects of these agents. Consequently, in such cases, treatment decisions should be carefully tailored, balancing possible therapeutic benefits against cardiovascular risks.

In our study, patients were categorized according to the presence of pre-existing heart disease; however, in everyday clinical practice, it is essential to recognize that cardiovascular disorders are heterogeneous and cannot be approached uniformly. Each patient should therefore be assessed individually to ensure that management strategies are adapted to the specific cardiac substrate and overall clinical context.

## 5. Limitations

The main limitation of this study lies in its design (i.e., observational, all-comer approach, electronic data collection), which may introduce relevant biases that could influence the results. It is also important to note that, to balance the two groups, patients were matched using variables known to influence prognosis. While this approach aims to reduce confounding factors, it may also make it more difficult to detect potential differences between groups.

Patients with a highly suspicious COVID-19 diagnosis who died before confirmation were included in this study, which raises the possibility of inadvertently including patients with other pathologies that could confound the results.

Given our study design, we cannot affirm that the observed results were attributable to corticosteroid treatment, as the influence of uncontrolled or unmeasured factors cannot be excluded.

There might be considerable variability in the indications and use of corticosteroids across different centers as well as an absence of data regarding the dose and duration of corticosteroid treatment, which represents a source of potential bias. Moreover, there are no data collected regarding some pharmacological therapies (e.g., antidiabetic drugs) that are frequently used in this subgroup of patients and could have prognostic impact.

A higher proportion of patients requiring invasive mechanical ventilation was present in the corticosteroid subgroup after propensity score matching, which may have obscured a potential beneficial effect of corticosteroid treatment. However, an additional Cox regression analysis did not demonstrate an association between invasive mechanical ventilation and mortality in our population.

Heart disease is a concept that includes a wide range of cardiovascular conditions. Different responses to corticosteroids across the different pathologies could influence the results.

## 6. Conclusions

This study provides an analysis of the effects of corticosteroid treatment in a cohort of patients with COVID-19 and pre-existing heart disease. The results showed no clear mortality benefit in this subgroup; however, no harmful effects were observed either, and a potential benefit in terms of hospital stay was appreciated. These findings highlight the need for further research to clarify the effects of corticosteroids in patients with pre-existing heart disease and to evaluate the safety of their use in this population, which is at higher risk of complications. Future studies should aim to determine whether specific subgroups of cardiac patients could benefit from tailored corticosteroid regimens and to identify potential biomarkers that may help guide individualized therapeutic decisions.

## Figures and Tables

**Table 1:** Post-propensity score matching clinical data.

Matched Population	Matched Population (796)	Corticosteroid Treatment (398)	Non-Corticosteroid Treatment (398)	*p*-Value
Age, years	76.4 ± 12.5	76.4 ± 12.7	76.3 ± 12.3	0.964
Male	533 (67.0%)	266 (66.8%)	267 (67.1%)	0.940
Hypertension	646 (81.2%)	323 (81.2%)	323 (81.2%)	1.000
Obesity	197 (24.7%)	95 (23.9%)	102 (25.6%)	0.565
Type 2 diabetes mellitus	268 (33.7%)	136 (34.2%)	132 (33.2%)	0.764
Dyslipidemia	420 (52.8%)	207 (52.0%)	213 (53.5%)	0.670
Smoking	53 (6.7%)	28 (7.0%)	25 (6.3%)	0.670
Chronic kidney disease	151 (19.0%)	74 (18.6%)	77 (19.3%)	0.786
Lung disease	344 (43.2%)	174 (43.7%)	170 (42.7%)	0.775
Cerebrovascular disease	139 (17.5%)	72 (18.1%)	67 (16.8%)	0.641
Liver disease	50 (6.3%)	25 (6.3%)	25 (6.3%)	1.000
History of cancer	154 (19.3%)	75 (18.8%)	79 (19.8%)	0.720
Immunosuppression	79 (9.9%)	41 (10.3%)	38 (9.5%)	0.722
Previous vaccination	122 (47.7%)	54 (41.9%)	68 (53.5%)	0.061
Anticoagulation therapy	314 (39.4%)	164 (41.2%)	150 (37.7%)	0.310
Aspirin	74 (30.2%)	33 (27.0%)	41 (33.3%)	0.284
ACEI/ARB	457 (57.4%)	233 (58.0%)	224 (56.3%)	0.519
Statins	91 (37.9%)	37 (30.3%)	54 (43.9%)	0.028
Respiratory failure during admission	629 (79.0%)	312 (78.4%)	317 (79.6%)	0.663
Heart failure during admission	165 (20.7%)	85 (21.4%)	80 (20.1%)	0.662
Kidney failure during admission	280 (35.2%)	144 (36.2%)	136 (34.2%)	0.553
Upper respiratory tract infection	133 (16.7%)	69 (17.3%)	64 (16.1%)	0.635
Pneumonia	744 (93.5%)	371 (93.2%)	373 (93.7%)	0.774
Sepsis	139 (17.5%)	72 (18.1%)	67 (16.8%)	0.641
SIRS	244 (30.7%)	133 (33.4%)	111 (27.9%)	0.910
ECMO or any circulatory support	68 (8.5%)	35 (8.8%)	33 (8.3%)	0.800
Non-invasive mechanical ventilation	144 (18.1%)	80 (20.1%)	64 (16.1%)	0.141
Invasive mechanical ventilation	85 (10.7%)	52 (13.1%)	33 (8.3%)	0.029
High-flow nasal cannula	223 (28.3%)	113 (28.4%)	112 (28.1%)	0.937
In-hospital relevant bleeding	53 (6.7%)	35 (8.8%)	18 (4.5%)	0.016
Hospital stay (days)	9.5 {5–16}	8.0 {4–14}	11 {7–18}	<0.001
In-hospital mortality	309 (38.8%)	164 (41.2%)	145 (36.4%)	0.167
All-cause mortality	333 (41.8%)	175 (44.0%)	158 (39.7%)	0.222
Follow-up time, days	16 [7.0–50.7]	17 [8–42.7]	16 [6–56.2]	0.450

Values are expressed as *n* (%), mean ± SD, or median [interquartile range]. ECMO: Extracorporeal Membrane Oxygenation; SIRS: Systemic Inflammatory Response Syndrome; ACEI: Angiotensin-Converting Enzyme Inhibitor; ARB: Angiotensin Receptor Blocker.

**Table 2:** Distribution of different types of heart disease.

Main Heart Disease	Matched Population (796)	Corticosteroid Treatment (398)	Non-Corticosteroid Treatment (398)
Arrhythmias	211 (26.5%)	111 (27.9%)	100 (25.1%)
Coronary	231 (29.0%)	109 (27.4%)	122 (30.7%)
Heart failure	81 (10.2%)	43 (10.8%)	38 (9.5%)
Valvular disease	67 (8.4%)	31 (7.8%)	36 (9.0%)
Combined	180 (22.6%)	90 (22.6%)	90 (22.6%)
Not recorded	26 (3.3%)	14 (3.5%)	12 (3.0%)

Values are expressed as *n* (%).

**Table 3:** Long COVID-19 symptoms according to corticosteroid treatment during hospitalization in patients with previous heart disease.

Long-Term COVID-19 Symptoms	Matched Population (487)	Corticosteroid Treatment (234)	Non-Corticosteroid Treatment (253)	*p*-Value
Long-Term COVID-19 Cardiovascular Traits				
Fatigue	107/245 (43.7%)	52/122 (42.6%)	55/123 (44.7%)	0.777
Dizziness	31/245 (12.7%)	20/122 (16.4%)	11/123 (8.9%)	0.079
Chest pain	24/245 (9.8%)	8/122 (6.6%)	16/123 (13.0%)	0.089
Acute coronary syndrome	8/245 (3.3%)	5/122 (4.1%)	3/123 (2.4%)	0.500
Palpitations	38/245 (15.5%)	23/122 (18.9%)	15/123 (12.2%)	0.150
Resting heart rate increase	18/245 (7.3%)	12/122 (9.8%)	6/123 (4.9%)	0.150
Syncope	7/245 (2.9%)	4/122 (3.3%)	3/123 (2.4%)	0.722
Any arrhythmia	40/245 (16.3%)	22/122 (18%)	18/123 (14.6%)	0.472
Atrial fibrillation	29/245 (11.8%)	15/122 (18%)	14/123 (11.4%)	0.825
Perimyocarditis	2/245 (0.8%)	2/122 (1.6%)	0/123 (0.0%)	0.247
Edema	18/245 (7.3%)	9/122 (7.4%)	9 (7.3%) /123 (0.0%)	0.986
Incident hypertension	5/245 (2.0%)	3/122 (2.5%)	2/123 (1.6%)	0.684
Left ventricular dysfunction	12/245 (4.9%)	8/122 (6.6%)	4/123 (3.3%)	0.254
Relevant bleeding	5/245 (2.0%)	5/122 (4.1%)	0/123 (0.0%)	0.029
Long-Term COVID-19 Neuro-Psychological Traits				
Headache	11/245 (4.5%)	6/122 (4.9%)	5/123 (4.1%)	0.747
Migraine	8/245 (3.3%)	5/122 (4.1%)	3/123 (2.4%)	0.500
Ageusia	18/245 (7.3%)	7/122 (5.7%)	11/123 (8.9%)	0.336
Cognitive disorder	19/245 (7.8%)	8/122 (6.6%)	11/123 (8.9%)	0.485
Anxiety	28/245 (11.4%)	18/122 (14.8%)	10/123 (8.1%)	0.103
Depression	20/245 (8.2%)	11/122 (9%)	9/123 (7.3%)	0.627
Tinnitus or hearing loss	14/245 (5.7%)	6/122 (4.9%)	8/123 (6.5%)	0.593
Sleep disorder	26/245 (10.6%)	13/122 (10.7%)	13/123 (10.6%)	0.982
Mood disorder	21/245 (8.6%)	13/122 (10.7%)	8/123 (6.5%)	0.246
Paranoia	17/245 (6.9%)	11/122 (9.0%)	6/123 (4.9%)	0.202
Other Long-Term COVID-19 Symptoms				
Cough	37/245 (15.1%)	20/122 (16.4%)	17/123 (13.8%)	0.574
Polypnea	16/245 (6.5%)	10/122 (8.2%)	6/123 (4.9%)	0.293
Sleep apnea	15/245 (6.1%)	9/122 (7.4%)	6/123 (4.9%)	0.415
Tongue involvement	5/245 (2.0%)	2/122 (1.6%)	3/123 (2.4%)	1.000
Digestive disorders	13/245 (5.3%)	5/122 (4.1%)	8/123 (6.5%)	0.401
Nausea and vomiting	7/245 (2.9%)	5/122 (4.1%)	2/123 (1.6%)	0.281
Intermittent fever	5/245 (2.0%)	4/122 (3.3%)	1/123 (0.8%)	0.213
Chills	6/245 (2.4%)	5/122 (4.1%)	1/123 (0.8%)	0.120
Hair loss	9/245 (3.7%)	5/122 (4.1%)	4/123 (3.3%)	0.749
Joint pain	14/245 (5.7%)	6/122 (4.9%)	8/123 (6.5%)	0.593
Myalgias	17/245 (6.9%)	3/122 (2%)	14/123 (11.4%)	0.06
Sweats	5/245 (2.0%)	2/122 (1.6%)	3/123 (2.4%)	1.000
Weight loss	23/245 (9.4%)	9/122 (7.4%)	14/123 (11.4%)	0.381
Anosmia	13/245 (5.3%)	6/122 (4.9%)	7/123 (5.7%)	0.787
Attention disorder	19/245 (7.8%)	10/122 (8.2%)	9/123 (7.3%)	0.797
Memory loss	24/245 (9.8%)	12/122 (9.8%)	12/123 (9.8%)	0.983
Follow-up time, days	35 [15–361]	31.5 [15–311]	36 [14–378]	0.450

Values are expressed as *n* (%), mean ± SD, or median [interquartile range]. Data was calculated using maximum number of subjects available for each variable.

## Data Availability

The data that support the findings of this study are derived from the international HOPE-2 registry. Due to privacy and ethical restrictions, the datasets are not publicly available. However, data may be made available from the corresponding authors upon reasonable request and subject to approval by the HOPE-2 registry steering committee.

## Supplementary Materials

The following supporting information can be downloaded at https://www.mdpi.com/article/10.3390/biomedicines13112665/s1, Table S1: Clinical data of patients with previous heart disease admitted to hospital due to COVID-19 infection.
