# Supplementary material for: The Long-Term Outcomes of Corticosteroid Use in COVID-19 Patients with Cardiovascular Disease: A Propensity-Matched Analysis from the Multi-Center International Prospective Registry (HOPE-2)
Source: Biomedicines. 2025 Oct 30;13(11):2665. doi: 10.3390/biomedicines13112665 (PMC12650574; doi:10.3390/biomedicines13112665)
Supplement: Supplementary file 1 [file biomedicines-13-02665-s001.zip › biomedicines-3812721-supplementary.pdf]

SUPPLEMENTARY MATERIAL

Pre-PSM	Heart disease population (1188)	Corticosteroid treatment (453)	Non corticosteroid treatment (735)	p-Value
Age		76.10	75.39	0.362
Male	771 (64.9%)	299 (66.0%)	472 (64.2%)	0.531
Hypertension	947 (79.7%)	369 (81.5%)	578 (78.6%)	0.241
Obesity	301 (25.3%)	108 (23.8%)	193 (26.3%)	0.352
Type 2 Diabetes Mellitus	371 (31.2%)	151 (33.3%)	220 (29.9%)	0.219
Dyslipidemia	624 (52.5%)	238 (52.5%)	386 (52.5%)	0.994
Smoking	100 (8.4%)	32 (7.1%)	68 (9.3%)	0.187
Chronic Kidney Disease	194 (16.3%)	89 (19.6%)	105 (14.3%)	**0.015**
Lung Disease	458 (38.6%)	200 (44.2%)	258 (35.1%)	**0.02**
Cerebrovascular Disease	202 (17.0%)	81 (17.9%)	121 (16.5%)	0.527
Liver Disease	69 (5.8%)	29 (6.4%)	40 (5.4%)	0.492
History of cancer	212 (17.8%)	89 (19.6%)	123 (16.7%)	0.203
Immunosuppression	108 (9.1%)	61 (13.5%)	47 (6.4%)	**0.000**
Respiratory failure during admission	764 (64.3%)	364 (80.4%)	400 (54.4%)	**0.000**
Heart failure during admission	234 (19.7%)	96 (21.2%	138 (18.8%)	0.309
Kidney failure during admission	347 (29.2%	175 (38.6%)	172 (23.4%)	**0.000**
Upper respiratory tract infection	172 (14.5%)	77 (17%)	95 (12.9%)	0.053
Pneumonia	1007 (84.8%)	426 (94%)	581 (79%)	**0.000**
Sepsis	171 (14.4%)	89 (19.6%)	82 (11.2%)	**0.000**
SIRS (Systemic Inflamatory Response Syndrome)	294 (24.7%)	179 (39.5%)	115 (15.6%)	**0.000**

Table S1: Clinical data of patients with previous heart disease admitted to hospital due to COVID-19 infection.
